# Developing a predictive tool for psychological well-being among Chinese adolescents in the presence of missing data

**DOI:** 10.1186/1471-2288-11-119

**Published:** 2011-08-19

**Authors:** Henry S Lynn, Bill Y Tsang

**Affiliations:** 1 Department of Biostatistics, School of Public Health, Key Laboratory on Public Health Safety of the Ministry of Education, Fudan University, Shanghai 200032, China; 2 The Youth Foundation, Breakthrough Centre, Hong Kong, China

**Keywords:** Missing data, Multiple imputation, Psychological maladjustment, Predictive tool

## Abstract

**Background:**

Multi-dimensional behavioral rating scales like the CBCL and YSR are available for diagnosing psychosocial maladjustment in adolescents, but these are unsuitable for large-scale usage since they are time-consuming and their many sensitive questions often lead to missing data. This research applies multiple imputation to tackle the effects of missing data in order to develop a simple questionnaire-based predictive instrument for psychosocial maladjustment.

**Methods:**

Questionnaires from 2919 Chinese sixth graders in 21 schools were collected, but 86% of the students were missing one or more of the variables for analysis. Fifteen (10 training, 5 validation) samples were imputed using multivariate imputation chain equations. A ten-variable instrument was constructed by applying stepwise variable selection algorithms to the training samples, and its predictive performance was evaluated on the validation samples.

**Results:**

The instrument had an AUC of 0.75 (95% CI: 0.73 to 0.78) and a calibration slope of 0.98 (95% CI: 0.86 to 1.09). The prevalence of psychosocial maladjustment was 18%. If a score of > 1 was used to define a negative test, then 80% of the students would be classified as negative. The resulting test had a diagnostic odds ratio of 5.64 (95% CI: 4.39 to 7.24), with negative and positive predictive values of 88% and 43%, and negative and positive likelihood ratios of 0.61 and 3.41, respectively.

**Conclusions:**

Multiple imputation together with internal validation provided a simple method for deriving a predictive instrument in the presence of missing data. The instrument's high negative predictive value implies that in populations with similar prevalences of psychosocial maladjustment test-negative students can be confidently excluded as being normal, thus saving 80% of the resources for confirmatory psychological testing.

## Background

China has undergone rapid urbanization and economic development in the past three decades with its urban population increasing from 18% to 46% between 1978 and 2008 [1]. However, accompanied with these changes, there has also been a disintegration of traditional family and social-supportive networks (e.g. divorce rates have risen from 0.07% in 1990 to 0.17% in 2008 [1]), contributing to greater stress among children and adolescents [2]. In a meta-analysis of 40 studies, self-reported anxiety levels were observed to have increased 0.7 standard deviations from 1992 to 2005, and anxiety levels were positively correlated with the Gini coefficient, divorce rate, unemployment rate, and crime rate [3]. Another analysis reported urban living to be a risk factor for drug use and casual sex [4]. Depression, social problems, and substance abuse were more prevalent among adolescents lacking family and community social capital [5-7], and children of rural-to-urban migrant workers were more prone to separation anxiety and depression because of heightened parental-child conflicts and discrimination at school [8]. The ignorance or under-detection of the effects of such turmoil and stresses on adolescents can translate into serious societal problems, while early intervention can be effective and beneficial [9,10].

Different multi-dimensional rating scales have been developed for detecting adolescents with behavioral abnormalities [11,12], and the ones that have been most frequently used include the Achenbach System of Empirically Based Assessment [13], Conners' Comprehensive Behavior Rating Scales [14], Behavioral Assessment System for Children [15], and the Revised Behavior Problem Checklist [16]. These tools are however unsuitable for large-scale screening since they are time-consuming and susceptible to non-response due to their many sensitive questions. The latter drawback can lead to large quantities of missing data in the analysis, resulting not only in a major loss of statistical power but possibly biased results. Instead, an effective instrument should be simple to administer, easy to answer, specific to its target population, and minimize the number of sensitive questions with labeling effects. This research addresses these concerns by considering the effects of missing data when developing a simple indigenous predictive tool for a large cohort of Chinese adolescents to assess their psychological adjustment as measured by several multi-dimensional behavioral rating scales.

## Methods

### Participants

The grade 6 students from twenty-one middle schools in Shanghai, China, were recruited, and altogether 2919 students (out of 2956) participated in the study. The schools were chosen to cover the span of academic levels, with 6 schools belonging to level I (high), 10 schools belonging to level II (middle), and 5 schools belonging to level III (low). The parents of these students were also asked to fill out a psychological assessment of their children. However, two schools were unable to follow this part of the protocol, and likewise only 2229 parents participated in the survey. Ethics approval was obtained and granted by the Institutional Review Board of the Institute of Psychology, Chinese Academy of Sciences.

### Measurements

Psychosocial adjustment was assessed using the Chinese validated versions of the 113-item syndrome scale of the Child Behavior Checklist (CBCL), the 112-item syndrome scale of the Youth Symptom Rating (YSR) [17,18], and the Mental Health Inventory (MHI) [19]. Students were classified as having psychosocial maladjustment if he/she was above the gender specific 95^th ^percentile on either one of the three multi-component scales. The 95^th ^percentiles for the YSR and CBCL were based on normal values obtained from a Chinese reference population in Hong Kong [20], while the 95^th ^percentile for the MHI were based on the current sample of Shanghai students. The decision to define the outcome measure primarily using Achenbach's CBCL and YSR scales was due to their comprehensive assessment of behavioral problems, solid psychometric properties, ability to collect information from different sources, extensive use in clinical research and practice, and availability of Chinese translations and Chinese reference norms [20,21].

A Student Information Form (SIF) consisting of 80 questions was developed to obtain information on the demographic, familial, health, academic, and social supportive characteristics of the students. The questions were based on literature review of risk factors for adolescents' problems, and were solicited from a panel of psychiatrists, psychologists, social workers, school principals and teachers, parents, and epidemiologists. An earlier version, the Hong Kong Student Information Form, had been developed and used in the Hong Kong Understanding Adolescents Project [22-24].

### Data Analysis

The purpose of the analysis was to develop and validate a psychosocial maladjustment predictive tool using a small number of questions from the SIF, while considering the effects of missing data. A typical strategy of dealing with missing data is to simply eliminate any observation that has incomplete data, but this type of "listwise deletion" or "complete case analysis" can result in the lost of many observations and statistical power when the analysis involves multiple variables [25]. Moreover, biased estimates are produced unless the data are missing completely at random [26,27]; i.e. the observations that have missing values are a random sample of the entire cohort. In practice, the reason data are missing may be related to different variables collected in a study; e.g. depressed and anxious students may be less likely to complete a psychological survey. An appropriate analysis should then use these variables to model the likelihood of having missing data, and incorporate the uncertainty associated with the modeling process. In this study, multiple imputation (MI) [28] was used to address these two concerns, and the multivariate imputation by chained equations (MICE) [29-31] algorithm implemented by the IVEware software [32] was used to perform the imputations. Fifteen imputed datasets were generated; ten datasets were used to "train" or develop the prognostic model for psychosocial maladjustment, and the remaining five datasets were used to validate the model. The stepwise variable selection method in logistic regression (with entry and exit cutoffs both set at *p *= 0.05) was used to select the SIF variables associated with psychosocial maladjustment for each of the ten training datasets. SIF variables selected with at least 70% frequency in the ten regression models were then combined into an additive SIF score using the mean of the regression coefficients of the SIF variables as weights. The area under the receiver-operating characteristic curve (AUC) and the calibration slope (i.e. the regression coefficient in the logistic regression of psychosocial maladjustment on SIF score) of this score were then calculated by combining the respective estimates across the five imputed validation datasets using PROC MIANALYZE [33]. The AUC serves as a discriminative measure of the score's ability in distinguishing between high versus low risk students. The calibration slope measures how well predicted probabilities agree with the observed probabilities, and equals one in the ideal case. Slopes of less than one imply over-optimistic predictions where low predictions are too low and high predictions are too high [34]. For illustrative purposes, a convenient cutoff was also chosen to dichotomize the score, and sensitivity, specificity, positive and negative predictive values, likelihood ratio of a positive test and negative test, and diagnostic odds ratio were calculated.

## Results

The characteristics of the cohort of Shanghai 6^th ^graders are presented in Table 1. The mean age was 11.9 years and 53% of them were male. Around 4.8% of the students felt unfavorably about their family, 6.7% had unfavorable opinions about their school, 3.8% had unfavorable ratings about their health, and 5.6% rated their social skills and social support environment as being unfavorable. The amount of missing data varied substantially across different variables. The median amount of missing data was 1.8% (range: 1.3% to 11.9%) among the SIF items, with 58.5% of the students missing at least one SIF item. The three case defining multi-component scales, CBCL, YSR, and MHI, had 59.3%, 27.1%, and 27.7% of their data missing, respectively, thus rendering 70% of the students not having a psychosocial maladjustment case definition. These students who had missing case definitions were more likely to be non-local students with lower academic rankings, less likely to argue with parents, and rated their social skills and social support environment more favorably. After applying MI the prevalence of psychosocial maladjustment was estimated to be 18.4% (95% CI: 16.5% to 20.2%), which was significantly lower than the 24.5% observed in the original sample.

**Table 1:** Characteristics of Cohort of 2919 Shanghai Students

Variable	N	Mean (SD)/Percentage	Percent Missing^c^
Age (years)	2915	11.9 (0.6)	0.1%
Male (%)	2918	53.3%	0.03%
CBCL	1188	13.6 (14.1)	59.3%
YSR	2129	24.4 (19.7)	27.1%
MHI	2110	173.5 (29.9)	27.7%
Psychosocial maladjustment ^a ^(%)	868	24.5%	70.3%
			
*SIF Summary Scores *^b^			
What is your overall feeling towards your family?	2879		1.4%
*1-3 (favorable)*	2451	85.1%	
*4 (neutral)*	291	10.1%	
*5-7 (not favorable)*	137	4.8%	
What is your overall feeling towards your school?	2869		1.7%
*1-3 (favorable)*	2311	80.5%	
*4 (neutral)*	366	12.8%	
*5-7 (not favorable)*	192	6.7%	
What is your assessment of your health?	2872		1.6%
*1-3 (favorable)*	2575	89.7%	
*4 (neutral)*	188	6.5%	
*5-7 (not favorable)*	109	3.8%	
What is your assessment of your social skills and social support environment?	2868		1.7%
*1-3 (favorable)*	2311	80.6%	
*4 (neutral)*	396	13.8%	
*5-7 (not favorable)*	161	5.6%	

^a ^Psychosocial maladjustment defined as a gender specific score above the 95^th ^percentile on either the CBCL, YSR, or MHI multi-component scales

^b ^SIF summary scores are rated on an integer scale of 1 (most favorable) to 7 (least favorable)

^c ^Calculated as a percentage out of 2919

Ten SIF variables were chosen based on applying the stepwise selection algorithm to the training datasets, and a SIF-Predictive Tool (SIF-PT) was constructed from these 10 variables (Table 2). Being male, having more positive feelings towards the family, spending less time on homework during weekends, having a good appetite, having mostly friends of the same sex, and having none or only one karaoke bar around the neighborhood all contributed positively to the SIF-PT. In contrast, being often ridiculed and ignored by classmates, regularly having difficulties in mathematics, and sleeping less than 8 hours a day were negatively associated with the SIF-PT. Higher SIF-PT scores were associated with a lower likelihood of psychosocial maladjustment. In the validation samples, the proportion of psychosocial maladjustment was 2.4% for students with SIF-PT scores greater than 3, but increased to 60.1% for those with scores of zero or less. The SIF-PT had an AUC of 0.75 (95% CI: 0.73 to 0.78) and its calibration slope was 0.98 (95% CI: 0.86 to 1.09) (Table 3). In order to contrast the MI approach with the listwise deletion approach of dealing with missing data, a stepwise logistic regression of psychosocial maladjustment on the SIF variables was also performed. This complete-case analysis had a sample size of only 415 and selected nine variables, three of which were included in the SIF-PT. The composite score constructed from this analysis had an AUC of 0.72 (95% CI: 0.69 to 0.76) and a calibration slope of 0.32 (95% CI: 0.26 to 0.38) in the validation samples.

**Table 2:** Definition of Student Information Form (SIF) Predictive Tool

SIF Item	Score
Gender	
*Male*	+0.60
*Female*	0
What is your overall feeling towards your family?	
1	+0.96
2	+0.94
3	+0.76
4 to 7	0
How often do your classmates ridicule you?	
*Regularly*	-1.00
*Sometimes*	-0.26
*Rarely*	-0.13
*Never*	0
How often do your classmates ignore you?	
*Regularly*	-0.92
*Sometimes*	-0.47
*Rarely*	-0.25
*Never*	0
How many hours do you spend doing your homework during weekends?	
*None/Less than one hour*	+0.41
*One to two hours*	+0.52
*Three to four hours*	+0.36
*Five hours or more*	0
Do you have difficulties in mathematics?	
*Regularly*	-1.03
*Sometimes*	-0.64
*Rarely*	-0.43
*Never*	0
How is your appetite?	
*Always good*	+0.78
*Somewhat good*	+0.73
*Not very good/Not good at all*	0
How many hours on average do you sleep per day?	
*Less than five/Five to six*	-0.80
*Seven to eight*	-0.16
*More than eight*	0
Are your friends mostly of the same sex?	
Yes	+0.41
No	0
How many karaoke bars are there around your neighborhood?	
*None*	+0.47
*One*	+0.70
*Two to three/Many*	0

**Table 3:** Characteristics of SIF Predictive Tool

	Training Samples		Validation Samples	
Measure	Estimate (SE)	95% CI	Estimate (SE)	95% CI
AUC	0.758 (0.017)	0.722 to 0.794	0.755 (0.014)	0.726 to 0.783
Calibration Slopeª	0.993 (0.076)	0.838 to 1.147	0.976 (0.059)	0.858 to 1.094
Sensitivity^b^	0.478 (0.030)	0.417 to 0.540	0.480 (0.025)	0.428 to 0.531
Specificity^b^	0.862 (0.008)	0.848 to 0.877	0.860 (0.007)	0.845 to 0.874
Positive Predictive Value^b^	0.441 (0.022)	0.397 to 0.485	0.431 (0.022)	0.388 to 0.473
Negative Predictive Value^b^	0.879 (0.011)	0.856 to 0.903	0.882 (0.008)	0.865 to 0.898
Likelihood Ratio of positive test^b^	3.478	2.879 to 4.200	3.414	2.918 to 3.993
Likelihood Ratio of negative test^b^	0.604	0.534 to 0.684	0.605	0.547 to 0.670
Diagnostic Odds Ratio^b, c^	5.755	4.241 to 7.810	5.638	4.390 to 7.241

ªCalibration slope is defined as the regression coefficient in the logistic regression of psychosocial maladjustment on the SIF Predictive Tool.

^b^Sensitivity, specificity, positive and negative predictive values, likelihood ratios of a positive and negative test, and the diagnostic odds ratio assume that a negative test is defined as when the SIF Predictive Tool score exceeds 1.

^c^Diagnostic odds ratio equals the likelihood ratio of a positive test divided by the likelihood ratio of a negative test.

When a SIF-PT score of > 1 was used to define a negative test, 79.8% of the students were classified as test negative, which is roughly the same percentage of students without psychosocial maladjustment. The resulting diagnostic test had a specificity of 86.0% (95% CI: 84.5% to 87.4%), sensitivity of 48.0% (95% CI: 42.8% to 53.1%), positive predictive value of 43.1% (95% CI: 38.9% to 47.3%), and negative predictive value of 88.2% (95% CI: 86.5% to 89.8%) in the validation samples (Table 3). Higher cutoffs can increase the sensitivity and reduce the number of false negatives, but the tradeoff is lower specificity and more false positives. For example, a cutoff of 2 yielded a test with a sensitivity of 83% but a specificity of 49%. In general, fixing a specific cutoff value is a difficult decision since the choice depends on the case prevalence in the target population and the costs of false positive and false negative classifications [35]. If equal costs and a 50% case prevalence were assumed, then the optimal cutoff can be obtained by maximizing the Youden index (or equivalently, minimizing the false positive plus false negative rates). The resulting test with this optimal cutoff of 1.6 had a sensitivity of 68.4% (95% CI: 63.7% to 73.1%), specificity of 67.9% (95% CI: 65.8% to 69.9%), positive predictive value of 32.0% (95% CI: 29.2% to 34.8%), and negative predictive value of 90.6% (95% CI: 89.0% to 92.3%) in the validation samples.

## Discussion

Missing data is a critical issue in this research. Although the ideal solution to dealing with missing data is not to have any, practical constraints make this difficult to comply. For example, two schools chose not to administer the CBCL since they think it would over-burden the parents, and thus 24% of the sample started without CBCL information. Also, there was a substantial amount of incomplete forms since many parents felt certain questions in the CBCL were intrusive. The response rate might have been improved if interviewers individually administered the CBCL to each parent, but due to limited resources the form could only be administered in a group setting. Ultimately, 70% of the outcome variable ended up missing. In such situations, the unguarded use of stepwise variable selection methods can select incorrect subsets of items and lead to inflated model performance. MI, however, can be used to account for the effects of missing data, and the fifteen imputed datasets were separated into training and validation sets to minimize the inclusion of irrelevant items and properly assess the performance of the SIF-PT. MI assumes that missing values may be dependent on observed variables but not on unobserved variables; i.e. missing at random [28], and the occurrence of missing data was verified to be associated with various student characteristics and behavior. Obviously, missing data may be related to unobserved variables; i.e. missing not at random [26]. However, appropriate analyses of data not missing at random highly depend on the choice of the postulated missing data model [36]. On the other hand, MI may still yield good estimates and standard errors even when the missing at random assumption is at fault [37]. Similar to previous studies [38,39], the MICE algorithm was employed for imputing the missing data as it provided greater stability and flexibility when handling many categorical variables. Amber, Omar, and Royston [29] also found that the MICE procedure yielded predictions with low bias and good coverage. Ten imputed datasets were used to develop the SIF-PT, and five additional imputed datasets were used to validate it in terms of its predictive ability. Clark and Altman [38] also developed their ovarian cancer prognostic model based on ten imputed datasets, and Heymans et al. [39] found that similar models were selected using ten versus one hundred imputations. A 70% threshold and a 0.05 significance level for selecting the SIF variables were adopted since the 70% cutoff has been shown to provide reasonable discriminative and calibrative properties [39], and the 5% significance level was found to be suitable for data where about half of the predictors were non-influential [40].

Although MI was successfully applied for analysis, its validity cannot be guaranteed especially with such large amounts of missing data as in the current study. Even further simulations can only provide anecdotal evidence since one can never ascertain the true values of the missing data for a specific study. However, the benefits of MI in this study lie in its theoretical and practical advantages over other common methods of handling missing data [28]. For example, a complete-case analysis discarded almost 86% of the cases, and the results are likely biased since the occurrence of missing data was dependent on variables like residence status, academic standing, relationship with parents, and quality of social support. The composite score obtained from this complete-case analysis had only three variables in common with the ten-variable SIF-PT. Its calibration slope estimate was also severely biased downward from one, indicating that it will be overly optimistic for prediction purposes.

In this study, the SIF-PT was internally validated using five imputed datasets. A good tool should be customized to the socio-cultural background of its target population in order to maximize predictability. For example, the small percentage (< 5%) of unfavorable attitudes towards one's family and the large percentage (21%) of students spending at least 5 hours doing homework during weekends are more characteristic of Asian children rather than those in North America or Europe. Moreover, the question concerning the number of karaoke bars around the child's neighborhood is distinctive to the type of social environment urban Chinese encounter. On the other hand, the tool should also have sufficient flexibility to encompass target groups beyond the original sampling frame; e.g. 7^th ^or 8^th ^graders, and other Chinese cities besides Shanghai. Admittedly, it is not easy to balance between these two competing objectives. Likewise, the external validity/generalizability of the SIF-PT to other Chinese cities awaits further research.

## Conclusions

Psychosocial maladjustment among adolescents can have serious consequences, and efforts at early detection and prevention are essential. Standardized rating scales like the CBCL and YSR are time-consuming, and their sensitive nature makes them susceptible to non-response. Such checklists are therefore inappropriate for large-scale evaluation, and the SIF Predictive Tool was developed to handle these deficiencies. Comprising of ten questions relating to the student's family, school, health, and social environment, it can be easily and quickly administered and is significantly associated with the risk of psychosocial maladjustment. In the validation samples, students with SIF-PT scores greater than three had a 2.4% risk of psychosocial maladjustment, while those with scores of zero or less showed a 25-fold increase in risk.

The SIF-PT's high negative predictive value implies that for populations with around 18% prevalence of psychosocial maladjustment one can forgo administering the CBCL, YSR, and MHI to test-negative students since they can be accurately predicted to be without psychosocial maladjustment. For example, psychological testing costs can be saved for 80% of the population who have SIF-PT scores greater than one since 88 out of 100 of these test-negative students can be correctly diagnosed as without psychosocial maladjustment. In general, for each individual student, a likelihood ratio can be derived from his/her SIF-PT score, and Bayes rule can be applied to compute the student's predictive probability of psychosocial maladjustment. For example, the likelihood ratios for SIF-PT scores ≤ 0, 0.1 to 1, 1.1 to 1.5, 1.6 to 2, 2.1 to 2.5, 2.5 to 3, and > 3 were 6.85, 2.46, 1.16, 0.83, 0.48, 0.22, and 0.11, respectively, in the validation samples. For a student with an ambivalent prior diagnosis (i.e., a 0.5 pre-test probability of psychosocial maladjustment), his/her post-test probability will be 0.87 and 0.71 for SIF-PT scores ≤ 0 and from 0.1 to 1, respectively, or 0.32, 0.18, and 0.10 for SIF-PT scores from 2.1 to 2.5, 2.5 to 3, and > 3, respectively. The former post-test probabilities support a psychosocial maladjustment diagnosis, while the latter post-test probabilities serve to exclude the possibility of maladjustment.

## Competing interests

The authors declare that they have no competing interests.

## Authors' contributions

All authors made substantial contribution to the manuscript. BYT participated in the design of this research and in the writing of the manuscript. HSL planned the design of this study, performed the analyses, and drafted the manuscript. All authors have read and approved the final manuscript.

## Pre-publication history

The pre-publication history for this paper can be accessed here:

http://www.biomedcentral.com/1471-2288/11/119/prepub
